# Bivalent activity of super-enhancer RNA *LINC02454* controls 3D chromatin structure and regulates glioma sensitivity to temozolomide

**DOI:** 10.1038/s41419-023-06392-w

**Published:** 2024-01-04

**Authors:** Tengfei Shi, Dianhao Guo, Yaoqiang Zheng, Wenbin Wang, Jinfang Bi, Anshun He, Sibo Fan, Guangsong Su, Xueyuan Zhao, Zhenhao Zhao, Yingjie Song, Shupeng Sun, Peng Li, Zhongfang Zhao, Jiandang Shi, Wange Lu, Lei Zhang

**Affiliations:** 1 State Key Laboratory of Medicinal Chemical Biology, Frontiers Science Center for Cell Responses, College of Life Sciences, Nankai University, 300071 Tianjin, China; 2 Department of Molecular Biology and Biochemistry, School of Clinical and Basic Medical Sciences, Shandong First Medical University & Shandong Academy of Medical Sciences, 250021 Jinan, shandong China; 3 Institute of Precision Medicine, The First Affiliated Hospital, Sun Yat-Sen University, 510030 Guangzhou, China; 4 Department of Neurosurgery, Tianjin Huanhu Hospital, School of Medicine, Nankai University, 6 Jizhao Road, 300350 Tianjin, China

**Keywords:** Cancer genomics, Cell death

## Abstract

Glioma cell sensitivity to temozolomide (TMZ) is critical for effective treatment and correlates with patient survival, although mechanisms underlying this activity are unclear. Here, we reveal a new mechanism used by glioma cells to modulate TMZ sensitivity via regulation of *SORBS2* and *DDR1* genes by super-enhancer RNA *LINC02454*. We report that *LINC02454* activity increases glioma cell TMZ sensitivity by maintaining long-range chromatin interactions between *SORBS2* and the *LINC02454* enhancer. By contrast, *LINC02454* activity also decreased glioma cell TMZ sensitivity by promoting *DDR1* expression. Our study suggests a bivalent function for super-enhancer RNA *LINC02454* in regulating glioma cell sensitivity to TMZ.

## Introduction

Glioma is the most common malignant brain tumor [1]. Glioblastoma (GBM), which accounts for 56.6% of all gliomas, has the highest rate of recurrence and a 5.6% 5-year survival rate [2]. Temozolomide (TMZ) is a chemotherapy drug widely used clinically as GBM treatment. However, development of TMZ resistance is a major problem that severely reduces drug efficacy. Regulation of GBM TMZ sensitivity is reportedly associated with genome repair systems in tumor cells, including O^6^-methylguanine-DNA methyltransferase (MGMT)-mediated demethylation, the base excision repair (BER) system, mismatch repair (MMR) and other activities [3, 4]. Previous studies also indicate that activities of glioma stem cells and cell autophagy alter TMZ sensitivity [5, 6]. Nonetheless, these discoveries have not yet been translated into novel, clinically-relevant approaches to GBM TMZ resistance.

Following development of chromosome conformation capture techniques, several groups reported that gene regulation activities based on 3D chromatin structure play a role in glioma cell sensitivity to TMZ [7–9]. In this process, gene regulatory elements, such as enhancers or silencers, exert long-range control of target genes via chromatin loops. Enhancer RNAs (eRNAs) are a subset of non-coding RNAs transcribed from an enhancer locus, and some are defined as long non-coding RNAs (lncRNAs) based on their length. eRNAs reportedly regulate enhancer function and thus play important roles in control of gene expression [10–12]. Other studies indicate that eRNAs function by increasing chromatin accessibility and binding to transcription factors [13–15]. However, few studies have assessed whether eRNAs function in a 3D gene regulatory network in a way that would alter glioma cell sensitivity to TMZ.

Super-enhancers (SEs) are large clusters of transcriptional enhancers that drive gene expression and often play key roles in cancer progression [16]. In this study, we identified a SE-derived long non-coding RNA, *LINC02454*, that regulates glioma TMZ sensitivity through maintenance of 3D chromatin structure. Our findings suggest that SE activity at the *LINC02454* locus increases glioma cell sensitivity to TMZ by regulating expression of *SORBS2* (Sorbin and SH3 domain containing 2) via long-range chromatin interactions, and that *LINC02454* maintains this chromatin loop. By contrast, we also show that *LINC02454* can reduce glioma cell TMZ sensitivity by promoting expression of *DDR1* (Discoidin domain receptor 1). Although other lncRNAs have been reported to function in glioma TMZ sensitivity [17, 18], our study reveals a new mechanism by which *LINC02454* and a corresponding SE co-regulate glioma sensitivity to TMZ by altering 3D chromatin structure and through bivalent *LINC02454* activities.

## Materials and methods

### Cell culture

U251 and U87 human glioblastoma cell lines were obtained from the National Infrastructure of Cell Line Resource (China). U251 and U87 cells were cultured in Dulbecco’s Modified Eagle’s medium (DMEM, Gibco) containing 10% fetal bovine serum (FBS, Bioind) and 1% penicillin/streptomycin (Gibco) at 37 °C, in 90% humidity and 5% CO_2_.

### Chromatin Immunoprecipitation and sequencing

ChIP-seq was carried out as reported with minor modifications [19]. 10^7^ cells were crosslinked 10 min with 1% formaldehyde (Thermo Fisher Scientific) at room temperature and the reaction was terminated by a 5 min incubation with a solution containing glycine (0.125 M) (Solarbio). After centrifugation at 4 °C, cells were washed twice with ice-cold PBS. Nuclear DNA precipitates were harvested in Farnham lysis buffer [50 mM (pH 7.5) tris-HCl, 150 mM NaCl, 5 mM EDTA, 0.5% NP-40, 1% Triton X-100, 1 mM phenylmethylsulfonyl fluoride and 1× protease inhibitor cocktail]. Nuclear DNA was then sonicated as a nuclear lysate (50 mM Tris-HCL,10 mM EDTA,1% SDS,1× protease inhibitor cocktail) to shear chromatin and obtain 200–500-bp DNA fragments. Sonicated lysates were immunoprecipitated at 4 °C with H3K27ac antibody (Abcam) and protein A/G agarose beads (Thermo Fisher Scientific) to enrich H3K27ac-bound DNA fragments. Purified ChIP DNA was used to prepare Illumina sequencing libraries and sequenced using an Illumina HiSeq 4000 system. Data analysis was performed using the GRCh37/hg19 reference genome. Super-enhancers were determined according to the Rank Ordering of Super Enhancers (ROSE) algorithm by using H3K27ac peaks [20]. In short, we used bowtie2 [21] to align ChIP-seq data to the human hg19 reference genome with default parameters. Then we used macs2 [22] to call peak with parameters “-f BAMPE -g hs”. We used ROSE [20] to identify SE with parameters “-g HG19 -s 20000 -t 2000”.

### RNA-seq and data analysis

Total RNA was extracted from glioma cells. The Illumina HiSeq 4000 platform was used to sequence barcoded RNA-seq libraries. HISAT2 [23] was used to map clean reads to the human reference genome (GRCh37/hg19). HTSeq [24] was used to calculate transcript abundance. DESeq2 [25] was used for read count normalization and to regularize log transformations. DAVID [26] (Database for Annotation, Visualization and Integrated Discovery) was used for Gene Ontology (GO) analysis and Kyoto Encyclopedia of Genes and Genomes (KEGG) analysis.

### Quantitative real-time PCR

Total RNA was extracted from glioma cells using TRIzol reagent (Life Technologies). cDNAs were obtained using the PrimerScript™ RT reagent Kit with gDNA Eraser (TaKaRa) for reverse transcription, according to the manufacturer’s instructions. The qPCR reaction was performed using Hieff^TM^ qPCR SYBR Green Master Mix (YEASEN) in the BioRad CFX Connect Real-Time system. Target gene expression levels were normalized to GAPDH and relative expression was quantified using the 2^−ΔΔCt^ method [27]. Primer sequences used in this study are shown in Table S1.

### Luciferase reporter assays

We PCR-amplified regions of the *LINC02454* SE showing the most significant H3K27ac enrichment (hg19_chr12:65,995,412–65,998,341) and cloned them into the pGL3 promoter vector (Promega). We then transfected 293 T cells with that plasmid plus the pRL-TK Renilla luciferase control vector (Promega) using Lipofectamine 3000 (Invitrogen). Cells were then harvested for a luciferase assay. Luciferase activity was measured on the SpectraMax i3x Multifunctional enzyme label instrument (Molecular Devices) using the dual luciferase Reporter Assay System (Promega).

### CRISPR/Cas9-mediated deletions

To delete the *LINC02454* SE, U251 glioma cells were transfected with plasmids containing Cas9 and guide RNAs targeting the SE region. Cell clones were genotyped, and two clones (KO#1 and KO#2) with homozygous deletion of the *LINC02454* SE were used for analyses. sgRNA sequences were designed using web-based tools at https://zlab.bio/guide-design-resources and are listed in Table S2.

### Lactate Dehydrogenase (LDH) assay

LDH in culture medium was evaluated using a LDH cytotoxicity kit (Promega). Briefly after cells were treated with TMZ or transfected with LNAs, siRNAs or overexpression plasmids, culture media were collected and LDH activity was assayed based on the manufacturer’s instructions. OD values at 490 nm were measured using a microplate reader. Relative TMZ cytotoxicity of each group was calculated as a percentage of the total amount of LDH released relative to a positive control included in the kit.

### Caspase 3/7 activity measurements

A Caspase-Glo 3/7 kit (Promega) was used to evaluate caspase 3/7 activities in U251 glioma cells based on the manufacturer’s instructions. Briefly, glioma cells in each group were harvested and incubated with 100 μL Caspase-Glo 3/7 reagent for 1 h in the dark. Luminescence was then measured at 485/530 nm using a microplate reader. Relative caspase 3/7 activity was calculated to evaluate fold-changes in samples treated with TMZ or transfected with LNAs, siRNAs, or overexpression plasmids relative to controls.

### Chromosome conformation capture (Capture-C) assay

Capture-C was performed as reported with minor modifications [28]. In brief, U251 glioma cells (1 × 10^7^) were harvested and used to generate a standard 3 C library with *Dpn*II (New England BioLabs). Then 3 C library DNA was sheared to 200–300-bp fragments by sonication. Pre-capture DNA libraries were prepared using the NEBNext DNA Library Kit (New England BioLabs) according to the manufacturer’s instructions. Biotinylated “bait” probes and streptavidin beads (Invitrogen) were used to enrich the “bait” and linked chromatin loci. Barcoded Capture-C libraries were sequenced as 150-bp paired-end reads using the Illumina HiSeq 4000 platform. Capture-C data were analyzed based on the pipeline proposed by Davies et al [28]. Two biotinylated DNA oligonucleotides were designed to match both ends of the H3K27ac enrichment region at the *LINC02454* locus in U251 and U87 glioma cells with the following sequence (5′-3′):

5′-Biotin-

GATCCCGAGAGCTTCCCGTGTGGGGGCGGAGGGTGGGGCAGAGCAGGATGTGTGCTTGGGTTTCACTTGGAAAAAACATACATCATATTGCAATATAATTTACTTTGAGATTTCAATTTGG-3′

5′-Biotin-

ACAATTGGCTGGAATCACAAGTGGCTTCTTTGCTCACTCTAGCGGTACTTCAGCTTTCATAAACAGCCGGTTCCTGGAGCTGTAAGGTTCCAGCACATCGGGGATTTCCCTTTGTTAGATC-3′

### Gene knock-down by LNAs (Locked Nucleic Acids)

Briefly, to knock-down *LINC02454*, cells were collected and placed in 6-well plates. Cells were then transfected with LNAs (QIAGEN) using Lipofectamine 3000 (Invitrogen) based on the manufacturer’s instructions. Seventy-two hrs later, cells were collected for RNA extraction, and KD efficiency was determined by qRT-PCR. LNA sequences are listed in Table S3.

### Gene overexpression

Candidate cDNAs were cloned into the pCDH-CMV-MCS-EF1-Puro expression vector (System Biosciences), and plasmids were used to transfect U251 cells with Lipofectamine 3000 (Invitrogen). Stably transfected cells were selected in media containing puromycin (2.5 μg/ml), and overexpression efficiency was determined by qRT-PCR.

### Gene knock-down by siRNA

Glioma cells were transfected with target and control siRNAs using Lipofectamine 3000 (Invitrogen) according to the manufacturer’s instructions. KD efficiency was determined by qRT-PCR 48–72 h after transfection. siRNAs were designed and synthesized by RiboBio Biotechnology Co., LTD., and the sequences are shown in Table S3.

### Chromatin isolation by RNA purification (ChIRP)-qPCR

ChIRP was performed as reported [29]. Antisense DNA probes complementary to *LINC02454* were biotin-labeled and ordered from Sangon Biotech. 10^7^ cells were cross-linked and fixed with 3% formaldehyde (Thermo Fisher Scientific), and the reaction was terminated by incubation with a solution containing glycine (0.125 M) (Solarbio). Cells were washed twice with PBS and lysed in lysis buffer. Cell lysates were then sonicated and incubated with biotinylated antisense DNA probes at 37 °C for 4 h. RNA and DNA from ChIRP samples were quantitated by qRT-PCR. qRT-PCR primer sequences are listed in Table S4, and the antisense DNA probe sequence is listed in Table S5.

### CRISPRa-mediated gene overexpression

A stable CRISPRa cell line was generated using lentiMPH v2 plasmid (Addgene). 293 T cells were transfected with pMDG.2, psPAX2 and lentiMPH v2 plasmid (Addgene). Lentiviral particles were then collected and used to transduce U251 cells, which were selected in 200 μg/ml hygromycin (Solarbio). sgRNAs were designed using an online tool (https://portals.broadinstitute.org/gppx/crispick/public) and ordered from Sangon Biotech, and their sequences are shown in Table S6. sgRNAs were cloned into lentiSAM v2 plasmid (Addgene) using *Bsm*BI (New England Biolabs). Lentiviral particles of pMDG.2, psPAX2 and lentiSAM v2 plasmid were generated and used to transduce U251 cells, which were selected in 6 μg/ml blasticidin (Solarbio) for about 7 days.

### Gene knock-down by shRNA

shRNAs were cloned into pSUPER-puro vector and used to transfect glioma cells with Lipofectamine 3000 (Invitrogen), according to the manufacturer’s instructions. Cells stably expressing shRNA were selected in puromycin (2.5 μg/ml) and KD efficiency was determined by qRT-PCR. qPCR primer sequences are shown in Supplementary Table S1. shRNAs were designed using an online tool (www.invivogen.com/sirnawizard/design.php) and synthesized by Sangon Biotech. shRNAs sequences are shown in Table S3.

### Survival data analysis

Glioma patients’ survival data were obtained from TCGA, and patient samples were divided into two groups based on gene expression levels. Samples with expression higher or lower than the median were marked as “high expression” or “low expression”, respectively. A *p* < 0.001 indicates a significant survival difference between the two groups.

### Statistical analysis

Data represent means ± S.E.M. Statistical analysis was performed using Student’s *t*-test. * *p* < 0.05, ** *p* < 0.01, *** *p* < 0.001.

### Published data used in this study

The following published datasets were used in this study. SRR444436 for U87 H3K27ac ChIP-seq [30]; SRR13238369 and SRR13238368 for NHA H3K27ac ChIP-seq [31]; SRR3627718 for U87 ATAC-seq [32]; SRR5583266 for U87 BRD4 ChIP-seq [33]; SRR444453 for U87 MED1 ChIP-seq [30]; and PRJNA479416 for RNA-seq of U251 cells treated for 0, 4, 9,12, and 16 days with TMZ [34].

## Results

### Identification of glioma-specific SE lncRNAs associated with glioma TMZ sensitivity

**Fig. 1:**
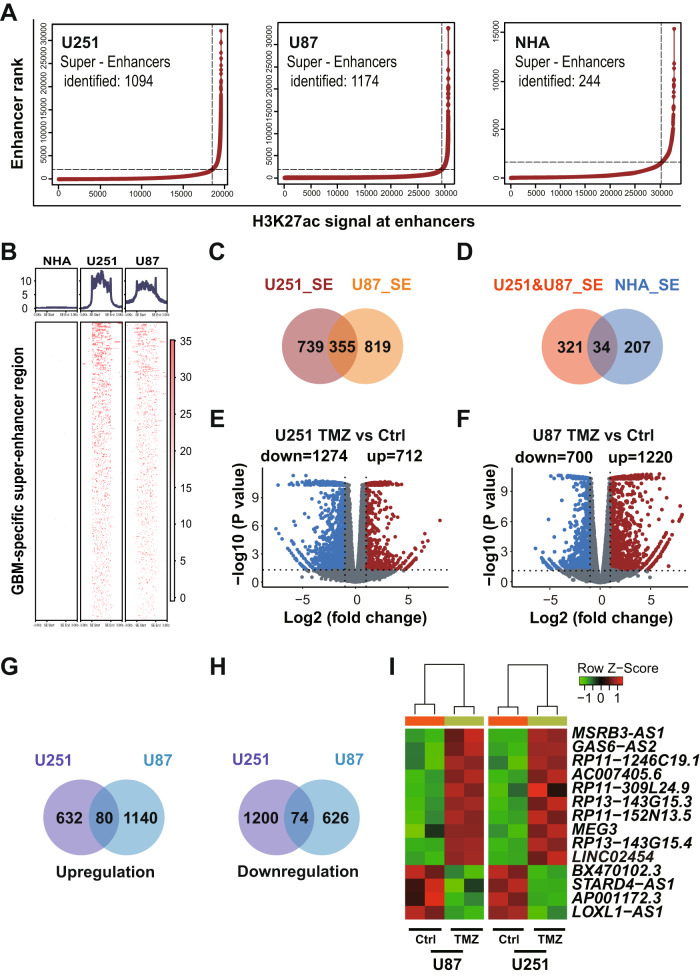
Identification of glioma-specific super-enhancer (SE) lncRNAs associated with glioma TMZ sensitivity. **A** Ranking of enhancers based on the H3K27ac signal at enhancers in glioma (U251 and U87) and astrocyte (NHA) lines. **B** H3K27ac signal of GBM-specific SE in U251, U87 and NHA cells. **C** Number of SEs in U251 and U87 cells. **D** Number of SEs in U251 and U87 cells compared to the number in NHA. Volcano plots of genes differentially expressed (FD ≥ 2 or ≤−2, *p* < 0.05) in U251 (**E**) and U87 (**F**) lines treated with TMZ (1 mM, 72 h), as compared with Ctrls. Number of up-regulated (**G**) and down-regulated (**H**) genes in U251 and U87 cells treated with TMZ (1 mM) for 72 h, as compared with Ctrls. **I** Heat map shows clustering of differentially expressed lncRNAs in U251 and U87 cells treated with TMZ (1 mM, 72 h), as compared to untreated Ctrl cells.

To identify enhancer lncRNAs that may regulate glioma cell TMZ sensitivity, we focused on lncRNAs derived from glioma-specific SEs. To identify such SEs, we used H3K27ac ChIP-seq data from glioma lines U251 and U87 and a normal human astrocyte line (NHA) (Fig. 1A, B). We identified 1094 and 1174 SEs in U251 and U87 cells, respectively, and 355 of those were common to both lines (Fig. 1C). Moreover, 34 SEs were identified in all three cell lines (Fig. 1D). Among SEs found in glioma lines, 321 were specific to U251 and U87 lines (Fig. 1D), while 207 SEs were specific to the NHA line (Fig. 1D). We then used data from the Cancer Genome Atlas (TCGA) and Genotype-Tissue Expression (GTEx) databases to assess transcript levels of genes adjacent to glioma-specific SEs. That analysis revealed significant changes in transcription of 52% of glioma-specific SE-associated genes, and 42.51% of those were significantly upregulated in glioma relative to normal brain tissue (Fig. S1A). Analysis of glioma-specific, SE-associated genes using the Kyoto Encyclopedia of Genes and Genomes (KEGG) indicated enrichment in focal adhesion and MAPK pathway factors (Fig. S1B), many previously linked to cancer cell proliferation, migration, apoptosis and chemoresistance [35–37].

**Fig. 2:**
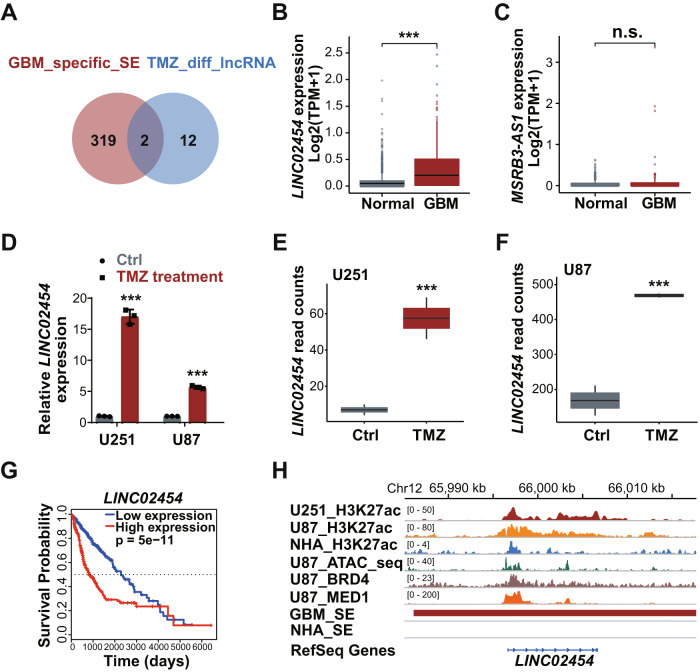
The super-enhancer (SE) lncRNA *LINC02454* is associated with glioma cell TMZ sensitivity. **A** Two lncRNAs that changed consistently in both TMZ-treated U251 and U87 cells (TMZ_diff_lncRNA) were located at glioma-specific SEs. Expression of *LINC02454* (**B**) and *MSRBS-AS1* (**C**) in GBM (166 cases) and normal brain (1157 cases) tissues. Gene Expression data are from TCGA and GTEx databases, ***p < 0.001. **D** qRT-PCR analysis of *LINC02454* RNA levels in TMZ-treated (1 mM, 72 h) and Ctrl cells. Data represent means ± S.E.M. of three independent experiments. ***p < 0.001, compared with Ctrl cells. **E**, **F** RNA-seq of read counts of *LINC02454* in Ctrl U251 and U87 cells treated with TMZ (1 mM, 72 h). **G** Analysis of survival data from glioma patients based on high and low *LINC02454* expression. **H** ATAC-seq and H3K27ac and BRD4 and MED1 ChIP of the *LINC02454* SE in glioma cells and astrocytes (NHA).

We then performed RNA-seq in TMZ-treated (1 mM, 72 h) U251 and U87 cells to monitor lncRNAs potentially associated with glioma TMZ sensitivity. That analysis identified 712 and 1220 genes (fold-change ≥ 2, *p* < 0.05) significantly up-regulated (Fig. 1E, F) and 1274 and 700 genes significantly down-regulated (fold-change ≤ −2, *p* < 0.05) in U251 and U87 cells, respectively (Fig. 1E, F). Eighty genes were up-regulated in both U251 and U87 lines, while 74 were down-regulated (Fig. 1G, H). Among genes that changed consistently in both cell lines, 14 were lncRNAs (Fig. 1I), and 2 of those, *LINC02454* and *MSRB3-AS1*, were located at glioma-specific SEs (Fig. 2A). Based on data from TCGA and GTEx databases, *LINC02454* expression in GBM samples was significantly higher than that in normal brain tissue (Fig. 2B), while *MSRB3-AS1* expression was comparable in GBM samples and normal brain samples (Fig. 2C), prompting our focus on *LINC02454*.

We next performed qRT-PCR analysis of U251 and U87 lines treated 72 h with TMZ (1 mM) and untreated controls. Relative to corresponding controls, TMZ-treated cells showed significantly upregulated *LINC02454* (Fig. 2D), consistent with RNA-seq data (Fig. 2E, F). Moreover, analysis of patient survival indicated that high *LINC02454* expression in tumor samples was significantly correlated with low survival probability (Fig. 2G). ChIP analysis also showed significant enrichment of H3K27ac signals at the *LINC02454* locus in U251 and U87 glioma cells relative to NHA astrocytes (Fig. 2H), suggesting that locus is a glioma-specific SE. In addition, ChIP-seq showed enrichment of SE-enriched transcriptional coactivators BRD4 and MED1 at the *LINC02454* locus in U87 glioma cells (Fig. 2H). Finally, ATAC-seq analysis indicated an open chromatin status of *LINC02454*-associated SE regions in glioma cells (Fig. 2H).

### *LINC02454* knock-down increases glioma cell sensitivity to TMZ

**Fig. 3:**
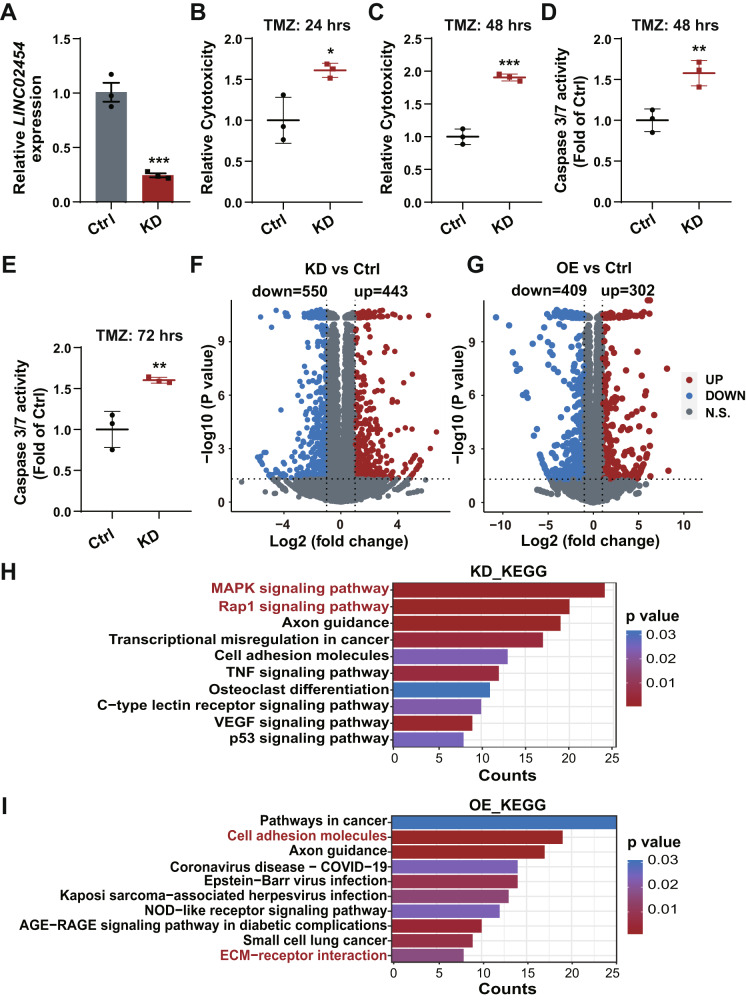
*LINC02454* KD decreases glioma cell TMZ sensitivity. **A** qRT-PCR was used to assess LNA KD efficiency in control (Ctrl) and *LINC02454* KD (KD) U251 glioma cells. Data represent means ± S.E.M. of three independent experiments. ***p < 0.001 compared with Ctrl. **B**, **C** LDH release levels from Ctrl and *LINC02454* KD cells treated with 1 mM TMZ for 24 h and 48 h. Data represent means ± S.E.M. of three independent experiments. *p < 0.05, ***p < 0.001 compared with Ctrl. **D**, **E** Caspase 3/7 activity in Ctrl and *LINC02454* KD cells treated with 1 mM TMZ for 48 h and 72 h. Data represent means ± S.E.M. of three independent experiments. **p < 0.01 compared with Ctrl. **F** Volcano plot depicting changes in gene expression in *LINC02454* KD and Ctrl cells. **G** Volcano plot depicting changes in gene expression in *LINC02454* overexpression (OE) and Ctrl cells. Functional analysis of genes differentially expressed in *LINC02454* KD (**H**) and *LINC02454* OE (**I**) cells relative to Ctrl cells.

To assess whether *LINC02454* activity regulates glioma cell sensitivity to TMZ, we transfected U251 cells with locked nucleic acids (LNAs) to knock-down (KD) *LINC02454*. qRT-PCR analysis of control and KD cells confirmed *LINC02454* downregulation to approximately a third of levels seen in control cells (Fig. 3A). We then evaluated lactate dehydrogenase (LDH) release and caspase 3/7 activity as indicators of cytotoxicity in both control and *LINC02454* KD U251 cells treated for various times with TMZ. Relative to TMZ-treated controls, *LINC02454* KD cells treated with TMZ (1 mM) showed significantly increased LDH release (Fig. 3B, C). Specifically, TMZ cytotoxicity in *LINC02454* KD cells increased to ~1.6-fold that of controls at 24 h (Fig. 3B) and to ~1.9-fold of controls at 48 h (Fig. 3C). Caspase 3/7 activities also markedly increased in TMZ-treated *LINC02454* KD relative to control glioma cells at 48 and 72 h time points (Fig. 3D, E). By contrast, *LINC02454* overexpression (OE) in U251 cells significantly decreased LDH release by 48 h of TMZ treatment relative to TMZ-treated controls (Fig. S2A, B). Overall, these findings suggest that *LINC02454* plays a role in TMZ sensitivity in glioma cells.

RNA-seq analysis of *LINC02454* KD and OE glioma cells revealed global changes in mRNA levels (Fig. 3F, G). Relative to controls, *LINC02454* KD cells showed significant down-regulation of 550 genes (fold-change ≤ - 2, *p* < 0.05) and up-regulation of 443 (fold-change ≥ 2, *p* < 0.05) (Fig. 3F). Moreover, relative to controls, *LINC02454* OE cells showed significant down-regulation of 409 genes (fold-change ≤ −2, *p* < 0.05) and up-regulation of 302 (fold-change ≥ 2, *p* < 0.05) (Fig. 3G). KEGG analysis of *LINC02454* KD glioma cells indicated that genes differentially expressed in *LINC02454* KD relative to control cells were significantly enriched in cancer progression-associated signaling pathways like MAPK and Rap1 (Fig. 3H). MAPK signaling is reportedly associated with regulation of glioma TMZ resistance [37]. Genes differentially expressed in *LINC02454* OE cells were significantly enriched in cell adhesion and signaling factors associated with ECM receptor interactions, which was found to be associated with TMZ resistance (Fig. 3I) [38]. Overall, these results suggest that *LINC02454* functions in glioma cell sensitivity to TMZ.

### Knock-out of the *LINC02454* super-enhancer decreases glioma cell sensitivity to TMZ

**Fig. 4:**
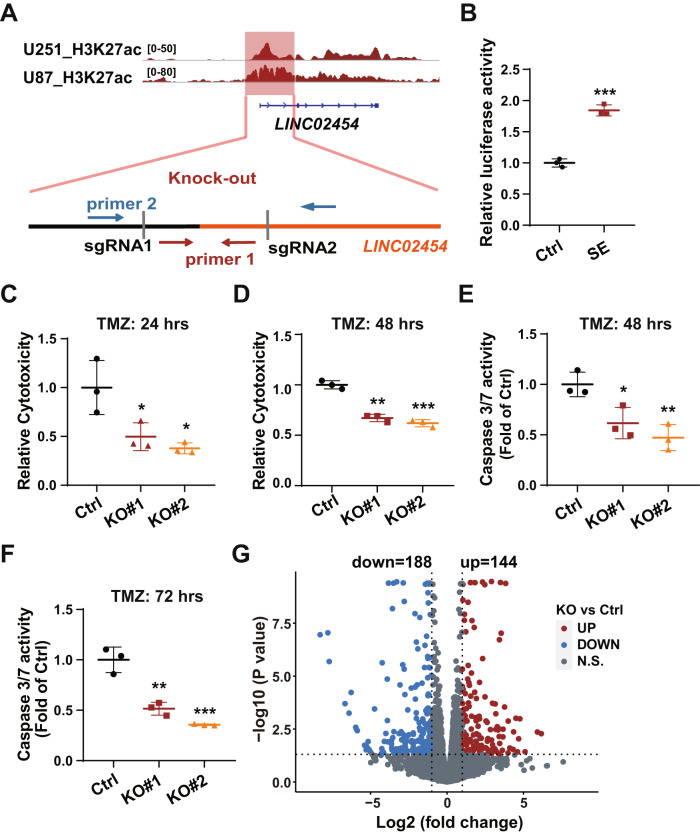
Knock-out of the *LINC02454* super-enhancer (SE) decreases glioma cell TMZ sensitivity. **A** A schematic showing the deleted *LINC02454* SE region and knock-out screening primers. **B** Luciferase reporter assay of Ctrl (reporter without the enhancer) and SE (reported plus *LINC02454* SE region) constructs performed in 293 T cells. Data represent means ± S.E.M. of three independent experiments, ***p < 0.001 compared with Ctrl. **C**, **D** LDH release levels in Ctrl and *LINC02454* SE KO (lines KO#1 and KO#2) cells treated with 1 mM TMZ for 24 h and 48 h. Data represent means ± S.E.M. of three independent experiments, *p < 0.05, **p < 0.01, ***p < 0.001 compared with Ctrl. Caspase 3/7 activity in Ctrl and *LINC02454* SE KO (lines KO#1 and KO#2) cells treated with 1 mM TMZ for 48 h (**E**) and 72 h (**F**). Data represent means ± S.E.M. of three independent experiments. *p < 0.05, **p < 0.01, ***p < 0.001 compared with Ctrl. **G** Volcano plot depicting changes in gene expression in *LINC02454* SE KO and control Ctrl cells.

Previous studies have reported coordinated functions of enhancers and corresponding enhancer lncRNAs in regulating physiological cellular processes [39, 40]. ChIP-seq data indicated H3K27ac enrichment at the *LINC02454* locus in glioma cells, which was identified as the *LINC02454* super-enhancer (*LINC02454* SE) (Fig. 4A). We then assessed enhancer activity using a luciferase reporter assay and found that the H3K27ac-enriched region has robust enhancer activity (Fig. 4B).

To investigate *LINC02454* SE function, we used the CRISPR/Cas9 system to knock out (KO) the H3K27ac enrichment region at that locus in U251 cells (Fig. 4A), resulting in KO#1 and KO#2 lines (Fig. S3A, B). We also evaluated LDH release and caspase 3/7 activity in TMZ-treated WT controls, KO#1 and KO#2 U251 lines. Compared with TMZ (1 mM)-treated WT controls, we observed a significant decrease in LDH release in TMZ (1 mM)-treated KO#1 and KO#2 cells (Fig. 4C, D). Specifically, TMZ cytotoxicity in KO#1 and KO#2 cells decreased to 40–50% of controls at 24 h TMZ treatment (Fig. 4C) and to 60–70% of controls at 48 h (Fig. 4D). Moreover, relative to control TMZ-treated U251 cells, caspase 3/7 activities markedly decreased in TMZ-treated KO#1 and KO#2 cells at 48 and 72 h time points (Fig. 4E, F), suggesting an overall decrease in glioma TMZ sensitivity in *LINC02454* SE KO cells.

RNA-seq analysis of WT controls and KO#1 and KO#2 U251 cells indicated global changes in mRNA levels in KO relative to control cells (Fig. 4G). Specifically, transcript levels of 332 genes significantly changed (fold-change ≥ 2 or ≤ −2, *p* < 0.05), including 188 down-regulated (fold-change ≤ −2, *p* < 0.05) and 144 up-regulated (fold-change ≥ 2, *p* < 0.05) in KO relative to WT U251 cells (Fig. 4G). Gene Ontology (GO) analysis showed down-regulated genes in cells were significantly enriched in categories related to cell adhesion, positive regulation of cell migration and regulation of phosphatidylinositol 3-kinase signaling associated with cancer progression (Fig. S3C). GO analysis of up-regulated genes in KO cells indicated significant enrichment in cancer progression-associated processes such as regulation of cell proliferation and cell death and Ras signaling (Fig. S3D). Down-regulated genes were significantly enriched in the PI3K-Akt signaling pathway, which is reportedly associated with glioma TMZ resistance (Fig. S3E) [41]. These results suggest that *LINC02454* SE KO induces transcriptional changes in several genes associated with glioma cell sensitivity to TMZ.

### *LINC02454* SE activity increases glioma cell sensitivity to TMZ by maintaining *SORBS2* expression via 3D chromatin structure

**Fig. 5:**
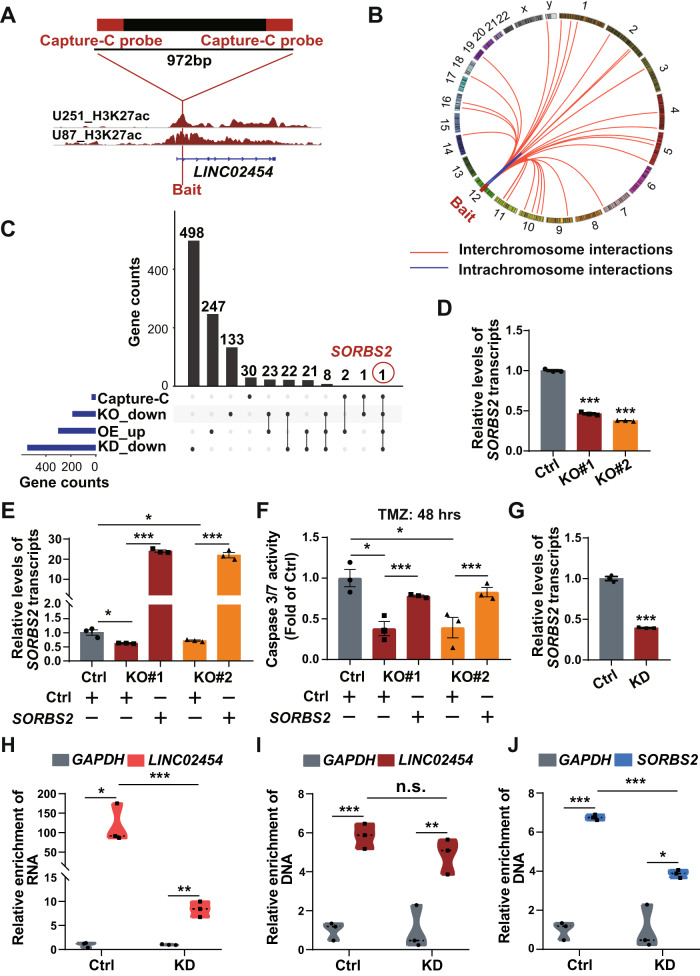
The *LINC02454* SE maintains *SORBS2* expression through 3D chromatin interactions and regulates glioma cell TMZ sensitivity. **A** The 972-bp Capture-C bait region and H3K27ac signals near the bait region. **B** Circos plot showing genome-wide intra- and inter-chromatin interactions, indicated by curves extending from the bait region. Intra- and inter-chromatin interactions are in blue and red, respectively. **C** Counts of genes that interact with the *LINC02454* SE (Capture-C), genes downregulated in *LINC02454* SE KO relative to Ctrl cells (KO_down), genes upregulated in *LINC02454* OE relative to Ctrl cells (OE_up), and genes downregulated in *LINC02454* KD compared with Ctrl cells (KD_down). **D** qRT-PCR analysis of *SORBS2* transcripts in Ctrl and *LINC02454* SE KO (lines KO#1, KO#2) cells. Data represent means ± S.E.M. of three independent experiments, ***p < 0.001 compared with Ctrl. **E** qRT-PCR verification of *SORBS2* overexpression in *LINC02454* SE KO cells. Data represent means ± S.E.M. of three independent experiments. *p < 0.05, ***p < 0.001. **F** Caspase 3/7 activity in Ctrl, *LINC02454* SE KO (lines KO#1, KO#2), and *SORBS2* OE/*LINC02454* SE KO (*SORBS2*) cells treated with 1 mM TMZ for 48 h. Data represent means ± S.E.M. of three independent experiments. *p < 0.05, ***p < 0.001. **G** qRT-PCR analysis of *SORBS2* transcript levels in Ctrl and *LINC02454* KD cells. Data represent means ± S.E.M. of three independent experiments. ***p < 0.001 compared with Ctrl. **H** ChIRP-qPCR analysis of *LINC02454* probe enrichment efficiency in Ctrl and *LINC02454* KD cells. Data represent means ± S.E.M. of three independent experiments. *p < 0.05, **p < 0.01, ***p < 0.001. ChIRP-qPCR analysis of *LINC02454* binding to the *LINC02454* SE (**I**) and the *SORBS2* locus (**J**) in Ctrl and *LINC02454* KD cells. Data represent means ± S.E.M. of three independent experiments. *p < 0.05, **p < 0.01, ***p < 0.001.

We then performed Capture-C, using a previously reported protocol [28], in U251 cells to define the 3D chromatin structure-mediated gene regulation network of the *LINC02454* SE, using the 972-bp H3K27ac enrichment region as Capture-C bait (Fig. 5A). Chromatin interactions between the *LINC02454* SE and target genes are shown in Circos plots [42] (Fig. 5B), in which lines represent interactions detected in two replicates (Fig. 5B). This analysis identified 34 genes in chromatin interaction regions potentially regulated by the *LINC02454* SE (Fig. 5B).

Combined analysis of both Capture-C and RNA-seq data in both SE KO cells and in *LINC02454* KD or OE cells indicated *SORBS2* downregulation in *LINC02454* KD and *LINC02454* SE KO cells and upregulation in *LINC02454* OE cells (Fig. 3F, G, Fig. 4G and Fig. 5C). Capture-C data also indicated a long-range interaction of *SORBS2*, which is located on chromosome 4, with the *LINC02454* SE (Fig. 5C). Consistent with RNA-seq results, qRT-PCR analysis confirmed down-regulation of *SORBS2* expression in *LINC02454* SE KO cells (Fig. 5D). We then transfected *LINC02454* SE KO lines with *SORBS2* OE vectors to establish stable lines and verified overexpression efficiency qRT-PCR (Fig. 5E). *SORBS2* OE in *LINC02454* SE KO cells significantly increased TMZ sensitivity in that line relative to *LINC02454* SE KO cells (Fig. 5E, F). We then assessed caspase 3/7 activities in TMZ-treated (1 mM, 48 h) control and *LINC02454* SE KO lines and in SE KO cells overexpressing *SORBS2*. Relative to the *LINC02454* SE KO line, caspase 3/7 activities markedly increased in *SORBS2*-overexpressing SE KO cells (Fig. 5F). Enhancer lncRNAs reportedly maintain interactions of corresponding enhancers with target genes [39, 40]. Accordingly, our RNA-seq data indicated parallel changes in expression of *SORBS2* and *LINC02454* (Fig. 5C), and qRT-PCR data showed significant *SORBS2* downregulation in *LINC02454* KD cells (Fig. 5G).

We next performed ChIRP-qPCR in control and *LINC02454* KD U251 cells to assess binding of *LINC02454* to the *LINC02454* SE and to the *SORBS2* locus. *LINC02454* RNA was effectively captured in control cells, while *LINC02454* RNA enrichment significantly decreased in *LINC02454* KD cells (Fig. 5H). *LINC02454* RNA was significantly enriched at the *LINC02454* SE in control cells, while those enrichment levels decreased in *LINC02454* KD cells (Fig. 5I). Moreover, *LINC02454* RNA was significantly enriched at the *SORBS2* locus in control cells but significantly decreased in *LINC02454* KD cells (Fig. 5J). These results indicate that *LINC02454* binds to the *LINC02454* SE and the *SORBS2* locus and suggest that *LINC02454* mediates interactions between the *LINC02454* SE and that locus. Previous studies have shown that enhancer lncRNAs activate target genes by interacting with Mediator complexes to create long-range chromatin loops [43, 44]. Mediator subunit MED1 was significantly enriched at *SORBS2* genomic loci interacting with the *LINC02454* SE (Fig. S4A), and *MED1* KD decreased *SORBS2* transcript levels (Fig. S4B). These results suggest that *MED1* and *LINC02454* together may mediate interaction of the *SORBS2* locus with the *LINC02454* SE.

Analysis of gene expression data from TCGA and GTEx databases showed significant downregulation of *SORBS2* transcripts in GBM and low grade glioma (LGG) relative to normal control samples (Fig. S5A, B), suggesting that *SORBS2* functions as a tumor suppressor in this context, an activity reported by others [45]. To verify *SORBS2* function in regulating glioma cell sensitivity to TMZ, we performed *SORBS2* OE followed by qRT-PCR analysis and confirmed a 170-fold upregulation in *SORBS2* transcript levels in glioma cells (Fig. S5C). We then assessed LDH release and caspase 3/7 activity in control and *SORBS2* OE glioma cells treated with TMZ. Compared with TMZ (1 mM)-treated control glioma cells, we observed significantly increased LDH release in similarly treated *SORBS2* OE cells (Fig. S5D). TMZ cytotoxicity in *SORBS2* OE cells significantly increased by 48 h of treatment (Fig. S5D). Consistently, caspase 3/7 activities in TMZ-treated *SORBS2* OE cells markedly increased by the 48 h time point, relative to comparably treated control cells (Fig. S5E). These results suggest overall that *SORBS2* upregulation increases glioma cell sensitivity to TMZ.

### *LINC02454* functions bivalently to regulate glioma cell sensitivity to TMZ

**Fig. 6:**
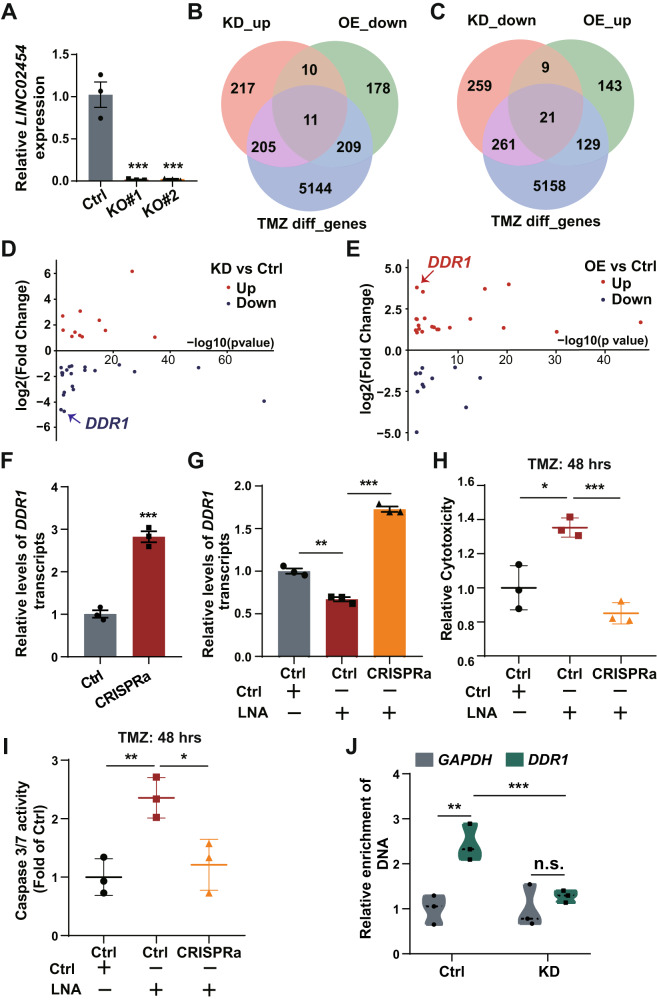
*LINC02454* decreases glioma cell TMZ sensitivity by promoting DDR1 expression. **A** qRT-PCR analysis of *LINC02454* expression of Ctrl and *LINC02454* SE KO (KO#1, KO#2) cells. Data represent means ± S.E.M. of three independent experiments, ***p < 0.001 compared with Ctrl. **B** Venn diagram showing intersection of three gene sets: KD_up (genes up-regulated in *LINC02454* KD cells), OE_down (genes down-regulated in *LINC02454* OE cells), TMZ diff_genes (genes differentially expressed in U251 cells after TMZ treatment for 4, 9, 12, and 16 days, compared with untreated U251 cells). **C** Venn diagram showing intersection of three gene sets: KD_down (genes downregulated genes in *LINC02454* KD relative to Ctrl cells), OE_up (genes upregulated genes in *LINC02454* OE relative to Ctrl cells), TMZ diff_genes (genes differentially expressed in U251 cells treated with TMZ for 4, 9, 12, and 16 days, compared with untreated U251 cells). Fold-change in transcript levels of 32 genes in *LINC02454* KD (**D**) and *LINC02454* OE (**E**) compared with Ctrl cells. **F** qRT-PCR analysis of *DDR1* mRNA levels in Ctrl and *DDR1*-CRISPRa cells (CRISPRa). Data represent means ± S.E.M. of three independent experiments, ***p < 0.001 compared with Ctrl. **G** qRT-PCR of *DDR1* mRNA levels in Ctrl and *DDR1*-CRISPRa cells transfected with LNA. Data represent means ± S.E.M. of three independent experiments, **p < 0.01, ***p < 0.001. **H** LDH release levels in Ctrl and *DDR1*-CRISPRa cells treated with LNA and 1 mM TMZ for 48 h. Data represent means ± S.E.M. of three independent experiments. *p < 0.05, ***p < 0.001. **I** Caspase 3/7 activity in Ctrl and *DDR1*-CRISPRa cells treated with LNA and 1 mM TMZ for 48 h. Data represent means ± S.E.M. of three independent experiments, *p < 0.05, **p < 0.01. **J** ChIRP-qPCR analysis of *LINC02454* binding to the *DDR1* locus in Ctrl and *LINC02454* KD cells. Data represent means ± S.E.M. of three independent experiments, **p < 0.01, ***p < 0.001.

Knock-out of the *LINC02454* SE in glioma cells downregulates *LINC02454* levels (Fig. 6A). However, *LINC02454* KD in glioma cells increased TMZ sensitivity (Fig. 3B–E), an outcome opposite to the decrease in TMZ sensitivity seen in *LINC02454* SE KO glioma cells. These findings suggest that *LINC02454* may regulate TMZ sensitivity in glioma cells by mechanisms other than maintaining chromatin interaction between the *LINC02454* SE and *SORBS2*.

To identify other genes regulated by *LINC02454* that govern glioma cell sensitivity to TMZ, we analyzed RNA-seq data of *LINC02454* KD and OE U251 glioma cells and U251 glioma cells treated with TMZ (50 µM) for 0, 4, 9,12, and 16 days [34] (Fig. 6B, C). That analysis indicated that transcript levels of 5569 genes changed with TMZ treatment. Among them, 11 were upregulated in *LINC02454* KD glioma cells and downregulated in *LINC02454* OE cells (Fig. 6B), while 21 were downregulated in *LINC02454* KD glioma cells and upregulated in *LINC02454* OE cells (Fig. 6C). Ranking these 32 genes based on fold-change in *LINC02454* KD or OE glioma cells, *DDR1* ranked in the top 3 both in *LINC02454* KD and OE glioma cells (Fig. 6D, E).

Gene expression data from TCGA and GTEx databases showed significant *DDR1* up-regulation in GBM and LGG relative to normal samples (Fig. S6A, B), suggesting *DDR1* has an oncogenic function. To confirm this function, we used the CRISPRa system to activate *DDR1* in U251 glioma cells. qRT-PCR data showed that CRISPRa-induction of *DDR1* significantly upregulated *DDR1* transcripts in control glioma cells and glioma cells that were transduced with *LINC02454* LNAs (Fig. 6F, G). *DDR1* upregulation decreased LDH release from glioma cells treated 48 h with TMZ (1 mM) (Fig. S6C). *DDR1* upregulation also significantly decreased caspase 3/7 activities in glioma cells treated 48 h with TMZ (1 mM) (Fig. S6D). We then evaluated LDH release and caspase 3/7 activity in control glioma cells and in CRISPRa glioma cells transduced with *LINC02454* LNAs and treated with TMZ. Compared with TMZ (1 mM, 48 h)-treated control glioma cells transduced with *LINC02454* LNA, we observed a significant decrease in LDH release in TMZ (1 mM, 48 h)-treated CRISPRa cells (Fig. 6H). Consistently, caspase 3/7 activities markedly decreased in 48 h TMZ (1 mM)-treated CRISPRa cells relative to control glioma cells transduced with *LINC02454* LNA (Fig. 6I). These data indicate that *DDR1* upregulation blocks the decrease in TMZ sensitivity seen in *LINC02454* KD glioma cells. ChIRP-qPCR results also showed significant enrichment of *LINC02454* at the *DDR1* locus in control but not *LINC02454* KD glioma cells (Fig. 6J), suggesting that *LINC02454* binds to that locus and regulates *DDR1* transcription.

## Discussion

There are several reports that lncRNAs play important roles in progression of several tumor types, including glioma [46–50], and function to regulate cancer cell proliferation, invasion, chemoresistance and metastasis [46, 51–53]. Some eRNAs are lncRNAs that function with a corresponding enhancer to regulate gene expression. Transcription of eRNAs is highly correlated with enhancer activity and corresponding promoter-driven gene transcription [10–12, 54]. For example, the eRNA *KLK3e* reportedly promotes chromatin interaction between its enhancer locus and *KLK2* to activate *KLK2* transcription in prostate cancer cells [55]. Lai et al. found that enhancer lncRNA functioned to establish or maintain chromatin interactions between enhancers and promoters, and lncRNA KD eliminated chromatin loops and those enhancer-promoter interactions [43].

Here, we show that the SE lncRNA *LINC02454* has bivalent and opposing functions in regulating glioma cell TMZ sensitivity. On one hand, *LINC02454* activity enhanced *SORBS2* expression by maintaining 3D chromatin interactions via the *LINC02454* SE, increasing glioma cell sensitivity to TMZ. On the other, *LINC02454* promoted *DDR1* expression to decrease glioma cell sensitivity to TMZ. Dynamic equilibrium of both of these functions may govern glioma progression by modulating responses to TMZ treatment, although further studies are needed to define *LINC02454* activities in specific contexts. Here, we also identified another lncRNA, *MSRB3-AS1*, as a potential SE lncRNA associated with TMZ sensitivity. However, *MSRB3-AS1* KD in glioma cells did not significantly alter their TMZ sensitivity (Fig. S7A–E). Compared with control, no significant change of LDH release was detected in *MSRB3-AS1* knock-down glioma cells that were treated with TMZ (1 mM) for 24 h and 48 h (Fig. S7B, C). Similarly, no significant change of caspase 3/7 acitivities were detected in *MSRB3-AS1* knock-down glioma cells that were treated with TMZ (1 mM) for 48 h and 72 h, relative to control (Fig. S7D, E).

In addition to synergizing with enhancers, lncRNAs reportedly also inhibit enhancer/target gene chromatin interactions. The lncRNA *Haunt* and its corresponding enhancer show opposing activities in regulating *HOXA* genes: *Haunt* inhibits chromatin interaction between its enhancer locus and the *HOXA* locus, downregulating *HOXA* gene expression [56]. In our study, 443 transcripts were significantly upregulated in *LINC02454* KD glioma cells. However, none of their gene loci showed a robust Capture-C signal with the *LINC02454* enhancer. By contrast, the *SORBS2* locus, which encodes a gene downregulated in *LINC02454* KD glioma cells, showed significant interaction with the *LINC02454* enhancer based on Capture-C data. Although further studies are needed to rule out the possibility that *LINC02454* inhibits chromatin interactions, our study suggests a synergistic function of *LINC02454* and the *LINC02454* SE in regulating *SORBS2* expression.

An important finding reported here is that *SORBS2* and *DDR1* are two major genes regulated by *LINC02454*, and that both regulate glioma cell sensitivity to TMZ. *SORBS2* encodes an RNA binding protein that regulates the actin cytoskeleton and cell movement [57]. *SORBS2* has been reported to function as a tumor suppressor in hepatocellular carcinoma, gastric cancer, pancreatic cancer, clear cell renal cell carcinoma and ovarian cancer [45, 58–61]. Mechanistically, SORBS2 protein stabilizes mRNAs with tumor suppressor function by binding to their 3′UTR [45, 60, 61]. Findings reported here support the idea that *SORBS2* expression is regulated by *LINC02454* and the *LINC02454* SE through long-range chromatin loops mediated by 3D chromatin structure. High levels of SORBS2 protein in glioma cells may stabilize mRNAs associated with tumor suppression and increase glioma cell TMZ sensitivity. DDR1 is a receptor tyrosine kinase that reportedly functions in development and progression of several cancer types [62–64]. In addition, DDR1 reportedly regulates tumor cell chemoresistance [65, 66]. We found that *LINC02454* KD downregulated *DDR1* transcription and increased glioma cell TMZ sensitivity, consistent with a previous study of GBM showing that inhibition of DDR1 combined with radiochemotherapy including TMZ increased TMZ sensitivity and prolonged patient survival [67]. DDR1 functions physiologically by activating MAPK, PI3K/Akt and other signaling pathways [68], many of which reportedly regulate TMZ resistance of glioma cells [37, 41]. In our study, KEGG analysis shown that *LINC02454* KD in glioma cells induced significant changes in MAPK signaling (Fig. 3H). Thus, *LINC02454* may regulate *DDR1* and alter glioma cell TMZ sensitivity through the MAPK pathway, although further studies are required for confirmation. Our findings also indicate that *SORBS2* and *DDR1* could be therapeutic targets to enhance glioma cell TMZ sensitivity. Also, modulation of corresponding regulatory mechanisms, such as long-range chromatin interactions at the *SORBS2* locus and via an enhancer mediated by *LINC02454*, could also enhance TMZ efficacy.

In conclusion, our study shows that *LINC02454* increases glioma cell TMZ sensitivity by maintaining chromatin interactions between the *SORBS2* gene and a *LINC02454* enhancer and at the same time can decrease TMZ sensitivity of glioma cells by promoting *DDR1* expression. These represent novel mechanisms used by *LINC02454* to regulate glioma cell TMZ sensitivity in two different contexts.

### Supplementary information

Supplementary Materials

Reproducibility checklist

## Data Availability

All sequencing data generated in this study have been submitted to the NCBI Gene Expression Omnibus (GEO; https://www.ncbi.nlm.nih.gov/geo/) under accession numbers GSE229600.
