# Supplementary material for: Bivalent activity of super-enhancer RNA LINC02454 controls 3D chromatin structure and regulates glioma sensitivity to temozolomide
Source: Cell Death Dis. 2024 Jan 4;15(1):6. doi: 10.1038/s41419-023-06392-w (PMC10766990; doi:10.1038/s41419-023-06392-w)
Supplement: Supplementary file 1 — Supplementary Materials [file 41419_2023_6392_MOESM1_ESM.pdf]

Supplementary Materials for

Bivalent activity of super-enhancer RNA *LINC02454* controls 3D chromatin structure and regulates glioma sensitivity to temozolomide

Tengfei Shi[1,#], Dianhao Guo[2,#], Yaoqiang Zheng[1], Wenbin Wang[1], Jinfang Bi[3], Anshun He[1], Sibo Fan[1], Guangsong Su[3], Xueyuan Zhao[1], Zhenhao Zhao[1], Yingjie Song[1], Shupeng Sun[4], Peng Li[1], Zhongfang Zhao[1], Jiandang Shi[1], Wange Lu[1,*], Lei Zhang[1,*]

*Corresponding author. Email: joyleizhang@nankai.edu.cn, wangelv@gmail.com

This PDF file includes:

Fig. S1 to S7

Table S1 to S6

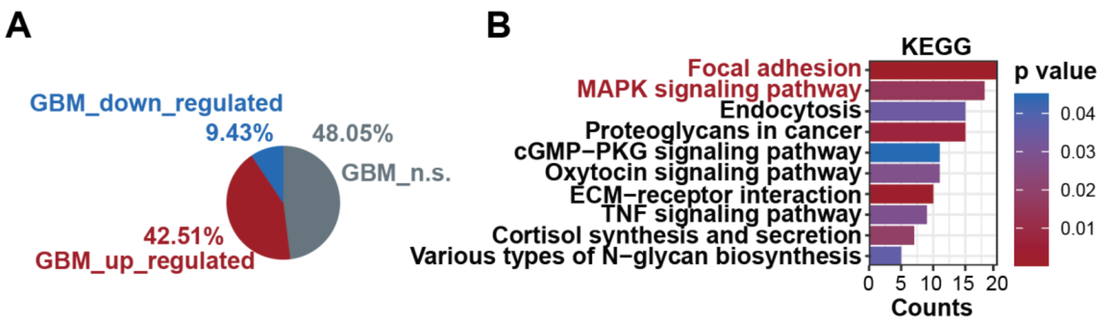

Fig. S1. Expression and functional analysis of GBM-specific, SE-related Genes. (**A**) Proportion of GBM-specific changes in SE-related gene expression in GBM relative to normal brain tissue from the TCGA database. (**B**) Functional analysis of GBM-specific, SE-related genes.

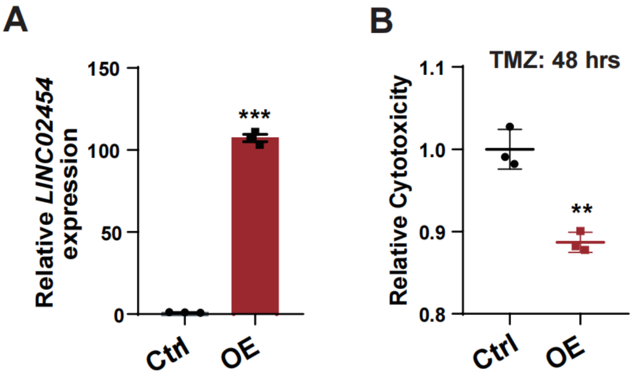

Fig. S2. *LINC02454* OE decreases glioma cell TMZ sensitivity. (**A**) qRT-PCR analysis of *LINC02454* in Ctrl and *LINC02454*-overexpressing (OE) cells. Data represent means ± S.E.M. of three independent experiments, ***p < 0.001 compared with Ctrl. (**B**) LDH release levels in Ctrl and *LINC02454* OE cells treated with 1 mM TMZ for 48 h. Data represent means ± S.E.M. of three independent experiments. **p < 0.01 compared with Ctrl.

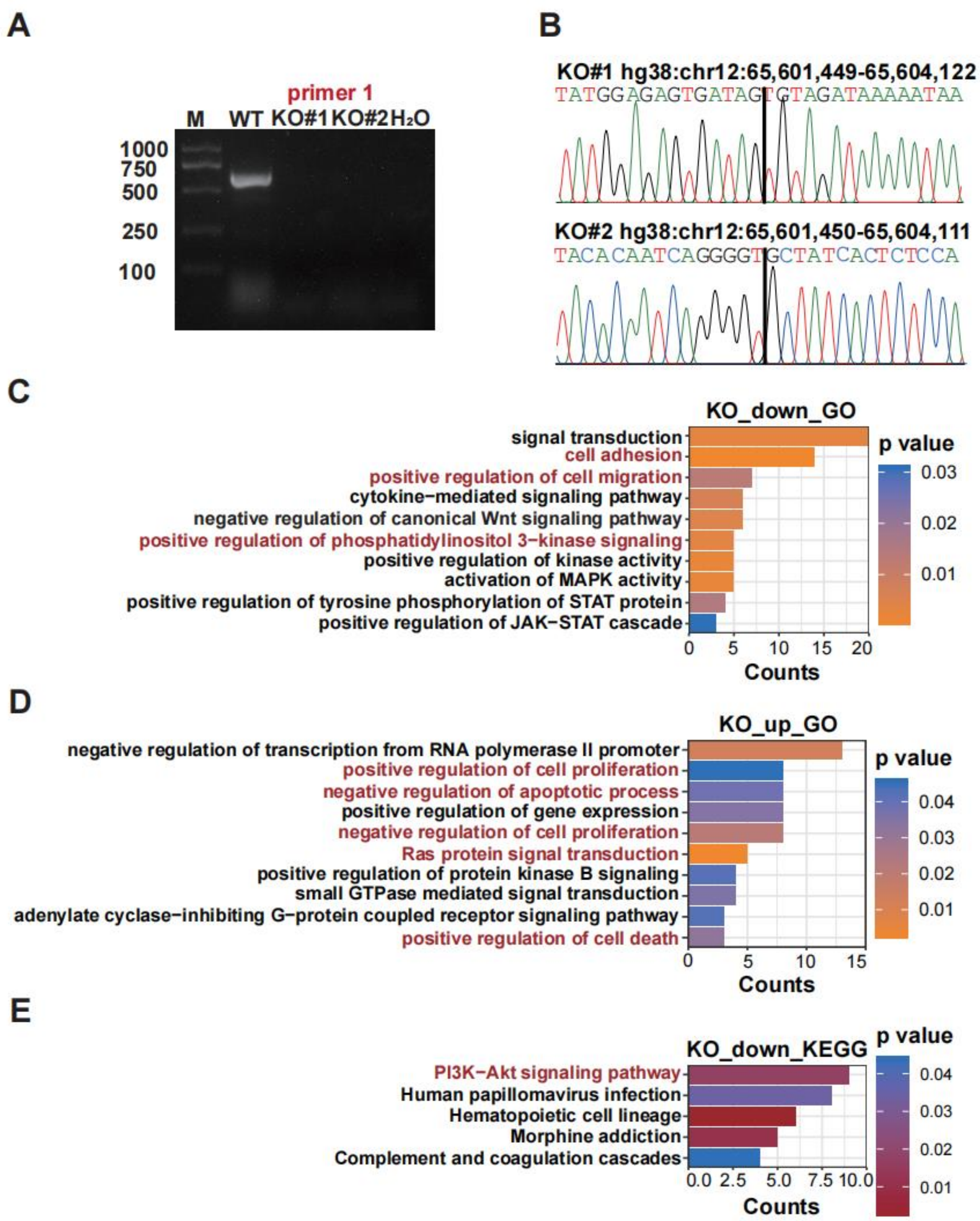

Fig. S3. CRISPR/Cas9–mediated deletion of the *LINC02454* SE. (**A**) Identification of *LINC02454* SE KO clones by PCR. WT lane shows PCR product of the intact SE region. PCR product indicates deletion of that region in clones KO#1 and KO#2. M, marker; H_2O, Negative control. (**B**) Sequencing of the *LINC02454* SE region in KO cells. (**C** and **D**) GO analysis of downregulated (**C**) and upregulated (**D**) genes in *LINC02454* SE KO relative to

Ctrl cells. (**E**) KEGG pathway analysis of genes downregulated in *LINC02454* SE KO relative to Ctrl cells.

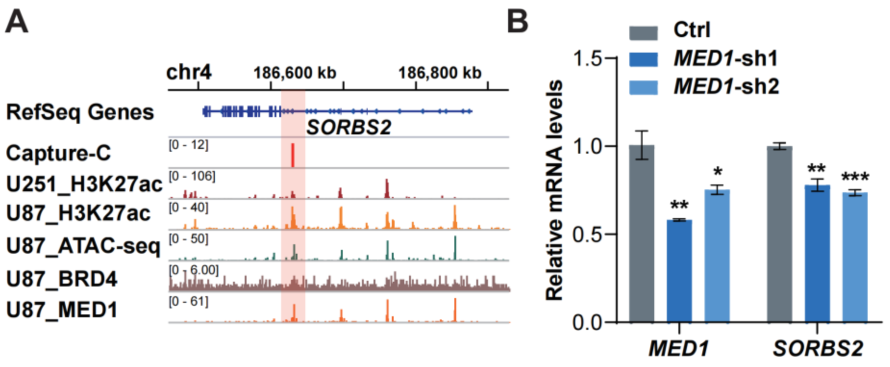

Fig. S4. ***MED1*** **functions in regulation of** ***SORBS2*** **expression by the** ***LINC02454*** **SE.** (**A**) Capture-C signal, ATAC-seq and H3K27ac, and BRD4 and MED1 ChIP-seq at the *SORBS2* locus. (**B**) qRT-PCR analysis of *SORBS2* transcript levels in Ctrl and *MED1* KD cells (*MED1*-sh1, -sh2). Data represent means ± S.E.M. of three independent experiments, *$p < 0.05$, **$p < 0.01$, ***$p < 0.001$ compared with Ctrl.

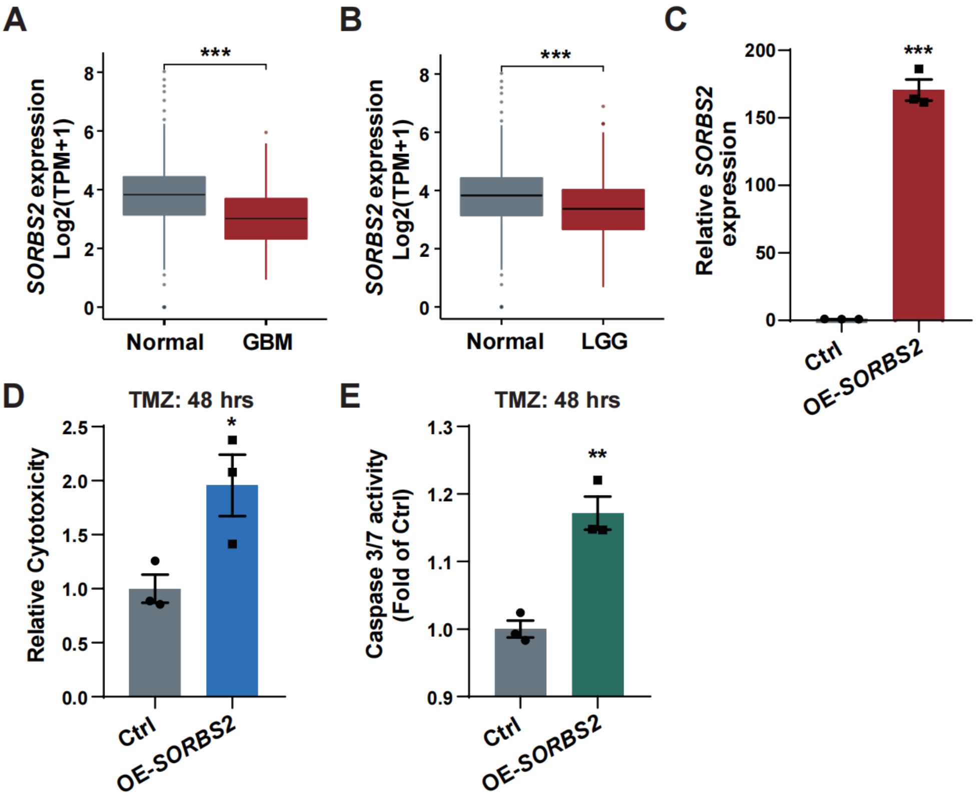

Fig. S5. *SORBS2* upregulation increases glioma cell TMZ sensitivity. (**A**) *SORBS2* expression in GBM (166 cases) and normal brain (1157 cases) tissues, ***$p < 0.001$. Data are from TCGA and GTEx databases. (**B**) *SORBS2* expression in low grade glioma (LGG) (523 cases) and normal brain (1157 cases) tissues, ***$p < 0.001$. (**C**) qRT-PCR analysis of *SORBS2* transcript levels in Ctrl and *SORBS2* overexpression (OE-*SORBS2*) cells. Data represent means ± S.E.M. of three independent experiments, ***$p < 0.001$ compared with Ctrl. (**D**) LDH release levels in Ctrl and *SORBS2* OE cells treated with 1 mM TMZ for 48 h. Data represent means ± S.E.M. of three independent experiments, *$p < 0.05$ compared with Ctrl. (**E**) Caspase 3/7 activity in Ctrl and *SORBS2* OE cells treated with 1 mM TMZ for 48 h. Data represent means ± S.E.M. of three independent experiments, **$p < 0.01$ compared with Ctrl.

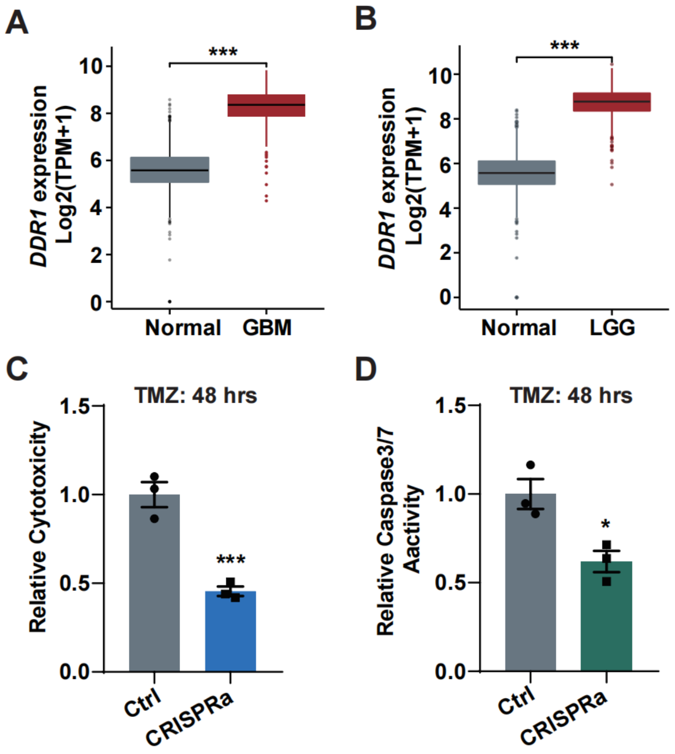

Fig. S6. *DDR1* upregulation decreases glioma cell TMZ sensitivity. (**A**) *DDR1* expression in GBM (166 cases) and normal brain tissue (1157 cases) samples, ****p* < 0.001. Data are from TCGA and GTEx databases. (**B**) *DDR1* expression in LGG patient (523 cases) and normal brain tissue control (1157 cases) samples, ****p* < 0.001. (**C**) LDH release levels in Ctrl and *DDR1*-CRISPRa cells treated with 1 mM TMZ for 48 h. Data represent means ± S.E.M. of three independent experiments, ****p* < 0.001 compared with Ctrl. (**D**) Caspase 3/7 activity in Ctrl and *DDR1*-CRISPRa cells with 1 mM TMZ for 48 h. Data represent means ± S.E.M. of three independent experiments, **p* < 0.05 compared with Ctrl.

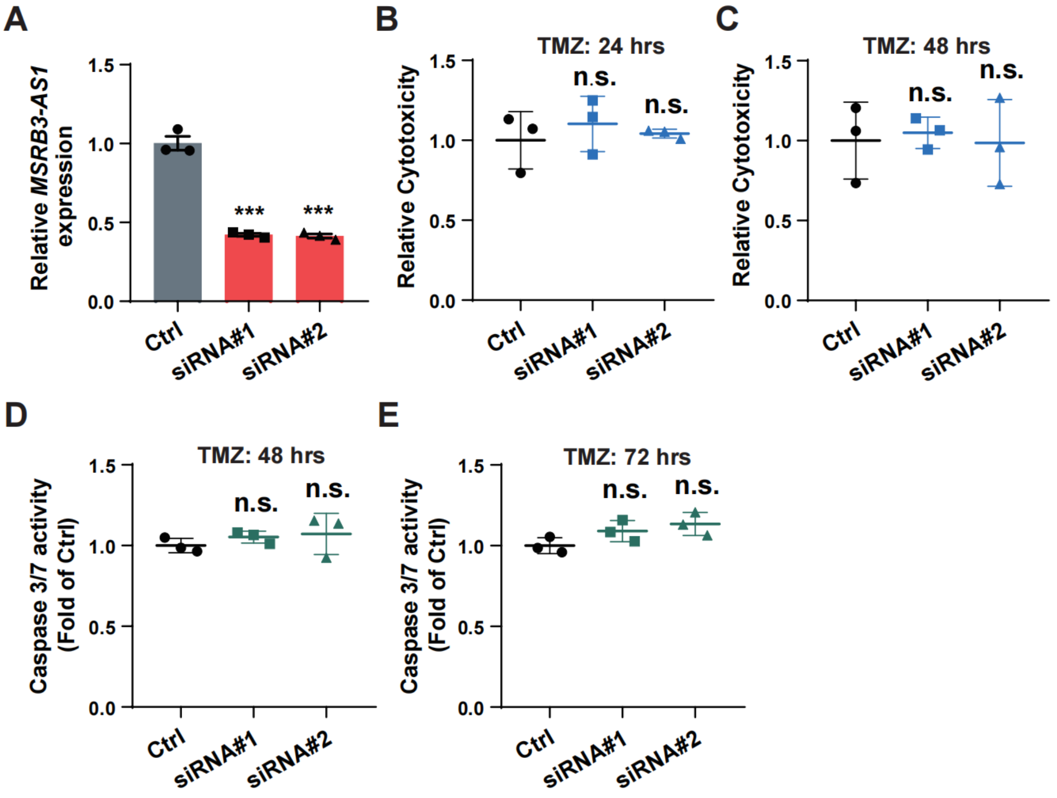

Fig. S7. *MSRB3-AS1* KD does not alter glioma cell TMZ sensitivity. (**A**) qRT-PCR analysis of RNA levels of lncRNA *MSRB3-AS1* in Ctrl and lncRNA *MSRB3-AS1* KD cells (siRNA#1, siRNA#2). Data represent means ± S.E.M. of three independent experiments, ***$p < 0.001$ compared with Ctrl. (**B** and **C**) LDH release levels in Ctrl and lncRNA *MSRB3-AS1* KD cells (siRNA#1, siRNA#2) treated with 1 mM TMZ for 24 h and 48 h. Data represent means ± S.E.M. of three independent experiments. (**D** and **E**) Caspase 3/7 activity in Ctrl and lncRNA *MSRB3-AS1* KD cells (siRNA#1, siRNA#2) treated with 1 mM TMZ for 48 h and 72 h. Data represent means ± S.E.M. of three independent experiments.

Table S1 Sequences of qRT-PCR primers

GAPDH-F	GTGGACCTGACCTGCCGTCT
GAPDH-R	GGAGGAGTGGGTGTCGCTGT
LINC02454-F	GTCACAGTTCAAACGCTCG
LINC02454-R	TCTAGTCTCCATGCACAGC
DDR1-F	GTCAGGAGGTGATCTCAGG
DDR1-R	CATAGAGCTCTACCCGCAG
SORBS2-F	TCAAATAAGTTGGCTTCCAGTG
SORBS2-R	CTGGAGAGCTCCTTTATCTGAG
MED1-F	CAGTTGACAATAAATGGACCC
MED1-R	TCAAGAAGAAACAGGCAGG
MSRB3-AS1-F	GTGGTCAGGTGTATGGGATATG
MSRB3-AS1-R	TGACCAGGAGTCTTCTACTTACT

Table S2 Knockout sgRNAs

LINC02454-KO-sgRNA1-F	ATGGAGAGTGATAGCACTAC
LINC02454-KO-sgRNA1-R	GTAGTGCTATCACTCTCCAT
LINC02454-KO-sgRNA2-F	TATCTACACAATCAGGGGTT
LINC02454-KO-sgRNA2-R	AACCCCTGATTGTGTAGATA

Table S3 Knockdown primers

Negative control-LNA	AACACGTCTATACGC

LINC02454-LNA	ACTTCAGACAAATTAC
MSRB3-AS1-siRNA-1	TCATTTCACCAACCCAGAA
MSRB3-AS1-siRNA-2	GAAACAAGACCGAACAACT
MED1-sh1-F	GCCAGTGGCACTGAATGTTAC
MED1-sh1-R	GTAACATTCAGTGCCACTGGC
MED1-sh2-F	GCAGAACTGCACAGGAATTCC
MED1-sh2-R	GGAATTCCTGTGCAGTTCTGC

Table S4 ChIRP-qPCR primers

GAPDH-ChIRP-RNA-F	GTGGACCTGACCTGCCGTCT
GAPDH-ChIRP-RNA-R	GGAGGAGTGGGTGTCGCTGT
LINC02454-ChIRP-RNA-F	GTCACAGTTCAAACGCTCG
LINC02454-ChIRP-RNA-R	TCTAGTCTCCATGCACAGC
GAPDH-ChIRP-DNA-F	TCGGGCCAATCTCAGTCCCT
GAPDH-ChIRP-DNA-R	CTAGTAGCCGGGCCCTACTTTC
LINC02454-ChIRP-DNA-F	TGGCGCTCCACATCGAATT
LINC02454-ChIRP-DNA-R	TCTGTGGTTCTGACGCCAT
SORBS2-ChIRP-DNA-F	CATGAAGTCAGCGATGTG
SORBS2-ChIRP-DNA-R	GGCGTGATTCTCAGTCACC
DDR1-ChIRP-DNA-F	AGAAGTAGGAGGGGCGTCTT
DDR1-ChIRP-DNA-R	TCCCCAAACGAGAACCTCCA

Table S5 DNA probes of ChIRP-qPCR

LINC02454-ChIRP-probe1	5'Biotin-TTTGTTTACAATCTTTATAGGTAGGGAATGATAAATCACAGTTAGACTTCTCAGCTAAT-3'
LINC02454-ChIRP-probe2	5'-Biotin-AGGCTTCATTGTTGAACCACCAAGGGTTGCTCCCTCTGTTTGATGGGGACGAGAAACTT-3'
LINC02454-ChIRP-probe3	5'-Biotin-CCAACATTCTGAGTGGTGACTATGTTTGGCCACGCCTGTGTCATACAGAAAAAAAGCGG-3'
LINC02454-ChIRP-probe4	5'-Biotin-TTTGGTAAATCACCACCTGAAATGAAAGAGCAGTCACCAAGCAGTACACACCGGCTGCC-3'
LINC02454-ChIRP-probe5	5'-Biotin-AAGCACGTCAGTCTGGGCGGAACCACTCTGGCTCCTTTTCAGGCAAACCTCCTTTCCTC-3'
LINC02454-ChIRP-probe6	5'-Biotin-CTTTGGGGAGACCTTTTGTCACAATTTTCCTCCAGTCTGACATGTTCTCTGTGGGAATG-3'
LINC02454-ChIRP-probe7	5'-Biotin-ACTCTCTTCACAGTAGCTGTAGAATTTGAAGGGAGCCTGAAAGGCATGACCTTGGAAGC-3'

LINC02454-ChIRP-probe8	5'-Biotin-CTTCCATTTCTAGTCTCCATGCACAGCCACTTCCCTGAGTGCATTCCAAATCCCAGATG-3'
LINC02454-ChIRP-probe9	5'-Biotin-GTCAGGGTACGAGCGTTTGAACTGTGACACAATTGCCATGAAGAAATATAAGGGGCCCT-3'
LINC02454-ChIRP-probe10	5'-Biotin-CCCTAGTCCAGGAGGTGAGGAGGACGATGTTCAAGCTGGATTATTTCCATGCCATTTTC-3'

Table S6 CRISPRa sgRNAs

Ctrl-CRISPRa-F	CCCGAATCTCTATCGTGCGG
Ctrl-CRISPRa-R	CCGCACGATAGAGATTCGGG
SORBS2-CRISPRa-F:	ACACTTTCACTTATCATCCC
SORBS2-CRISPRa-R:	GGGATGATAAGTGAAAGTGTC
DDR1-CRISPRa-F	GCGCCGCCTCCCCCTCGCCT
DDR1-CRISPRa-R	AGGCGAGGGGGAGGCGGCGCC
